# Supplementary material for: Electrical Stimuli-Responsive Decomposition of Layer-by-Layer Films Composed of Polycations and TEMPO-Modified Poly(acrylic acid)
Source: Polymers (Basel). 2022 Dec 7;14(24):5349. doi: 10.3390/polym14245349 (PMC9782790; doi:10.3390/polym14245349)
Supplement: Supplementary file 1 [file polymers-14-05349-s001.zip › polymers-2056304-supplementary.pdf]

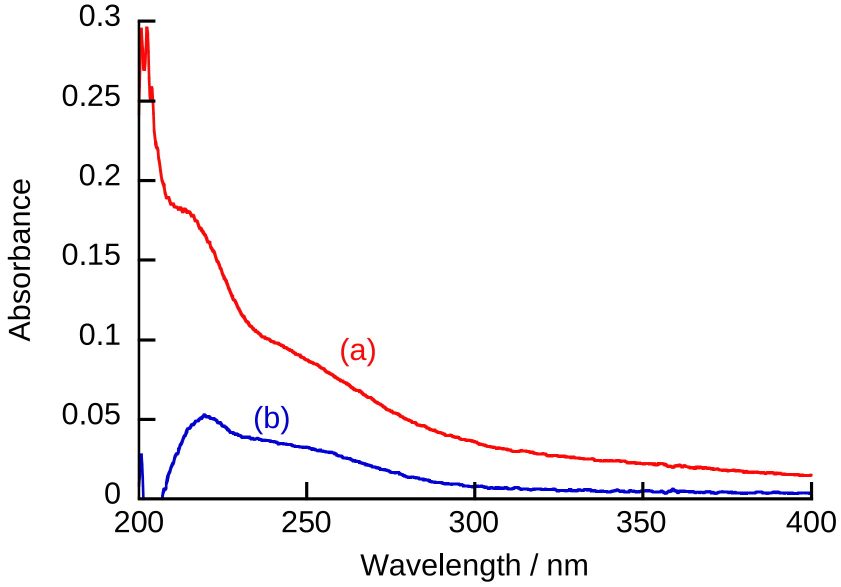

Figure S1. UV-vis absorption spectra for the (PEI/TEMPO-PAA)$_5$ film (a) before and (b) after exposure to 1 mM sodium hypochlorite solution (pH 10) for 30 min. A LbL film was prepared on the surface of a quartz slide. The (PEI/TEMPO-PAA)$_5$ films exposeed in sodium hypochlorite solution were rinsed with working buffer for 5 min.
